# A Rare Presentation of a Fibrous Breast Hamartoma Requiring Careful Imaging Assessment

**DOI:** 10.7759/cureus.98651

**Published:** 2025-12-07

**Authors:** Kento Tanaka, Shoji Oura

**Affiliations:** 1 Department of Surgery, Kishiwada Tokushukai Hospital, Kishiwada, JPN

**Keywords:** fibrous breast hamartoma, low internal echoes, low signals on t2-weighted images, no mass enhancement, no obvious fat findings on mammography

## Abstract

Breast hamartomas are benign disorders and are generally composed of breast-constituting pathological components, mainly fat components. A 68-year-old woman was found to have a left breast mass on computed tomography (CT) for coronary artery evaluation due to suspected angina pectoris. Mammography showed a well-defined oval mass, 2.7 cm in size, without obvious fat findings in the upper and outer quadrant of her left breast. Ultrasound showed a well-circumscribed mass with predominant low internal echoes, high internal echoes in very small areas, weakly enhanced posterior echoes, and no blood flow into and around the mass. Magnetic resonance imaging (MRI) of the breast mass showed low signals on T1-weighted images, predominant low signals on fat-suppressed T2-weighted images, and no enhancement through early-to-late-phase images on dynamic studies. The patient, therefore, underwent a core needle biopsy, which unfortunately only showed abundant fibrous components and no malignant cells, leading to no definitive pathological diagnosis. We, therefore, treated the breast mass with a lumpectomy to get both a pathological diagnosis and a cure of the presumed benign breast mass. The bisected mass had whitish cut surfaces except for the core needle biopsy-induced hemorrhage areas. Postoperative pathological study showed predominant fibrous components, a small amount of adipocytes, and sparse mammary duct-lobular structures in the fibrous components, leading to the diagnosis of fibrous breast hamartoma. The patient recovered uneventfully and is scheduled to receive annual mammography follow-ups. Diagnostic physicians should note that breast hamartomas can have predominant fibrous components.

## Introduction

Breasts can have various benign disorders such as intraductal papillomas and fibrocystic diseases [1]. Breast specialists often manage benign breast diseases not with surgery but with simple observation under the presumed diagnosis of benign diseases, even with no pathological diagnosis. On the other hand, breast surgeons sometimes treat benign breast diseases, e.g., fibroadenomas [2], with surgery when they become large or show rapid growth.

Breast hamartomas are benign disorders, have distinct borders of fibrous capsules, and are composed of breast-constituting pathological components with markedly different compositional ratios compared to those in the normal breast [3]. Depending on the amount of predominant pathological components, breast hamartomas have various subtypes, such as adenolipomas and chondrolipomas. However, breast hamartomas commonly have a large, if not the most abundant, amount of adipose tissue.

Well-demarcated breast hamartomas with abundant fat rarely pose challenges for physicians in the differential diagnosis of breast disorders [4]. In addition, diagnostic physicians seldom evaluate the correlation between the image and pathological findings of breast hamartomas due to the exceptional application of surgical intervention to them. In short, the vast majority of diagnostic physicians have never received pathological feedback for presumed breast hamartomas and therefore sometimes struggle to diagnose atypical breast hamartoma cases. We, herein, report an extremely rare case of fibrous breast hamartoma with characteristic image findings.

## Case presentation

**Figure 1:**
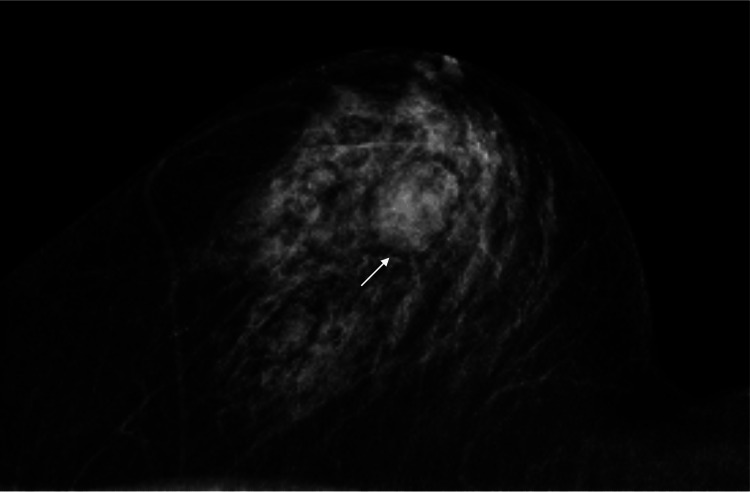
Mammography findings Mammography showed an oval mass (arrow) without obvious fat findings in the upper and outer quadrant of the patient's left breast.

A 68-year-old woman with no medical history, including breast diseases, was found to have a mass in her left breast on computed tomography (CT) for the coronary artery evaluation due to suspected angina pectoris and visited our hospital in July 2025. We palpated a round and smooth mass in her left breast. Mammography showed a well-defined oval mass, 2.7 cm in size, without obvious fat findings in the upper and outer quadrant of her left breast (Figure 1).

**Figure 2:**
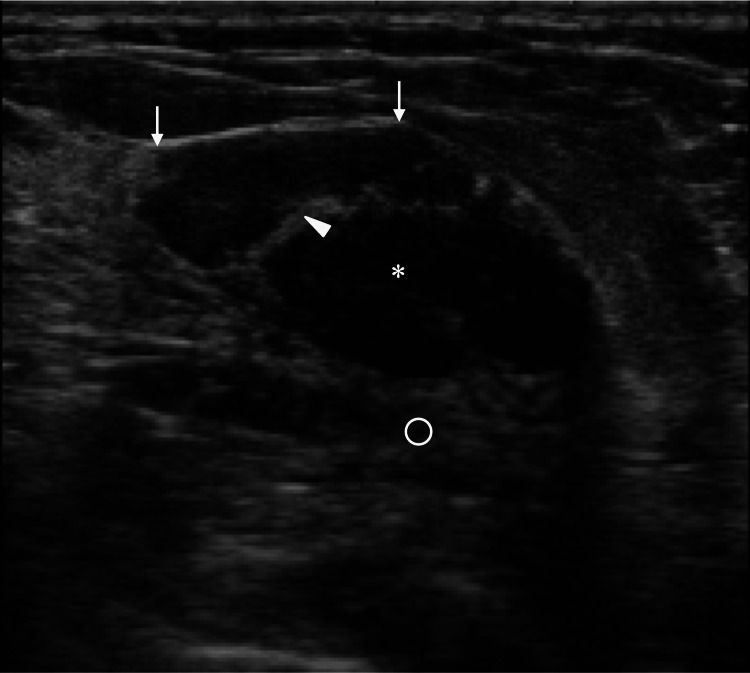
Ultrasound findings Ultrasound showed a well-circumscribed mass (asterisk) with predominantly low internal echoes, internal high echoes in very limited areas (arrowhead), and enhanced posterior echoes (open circle). Interfaces between the mass and the surrounding fat showed very strong high echoes at the mass margins parallel to the overlying skin (arrows).

Ultrasound showed a mass with predominant low internal echoes, high internal echoes in very small areas, weakly enhanced posterior echoes, focal strong high echoes at the interfaces between the mass and the adjacent fat, and no blood flow into and around the mass (Figure 2).

**Figure 3:**
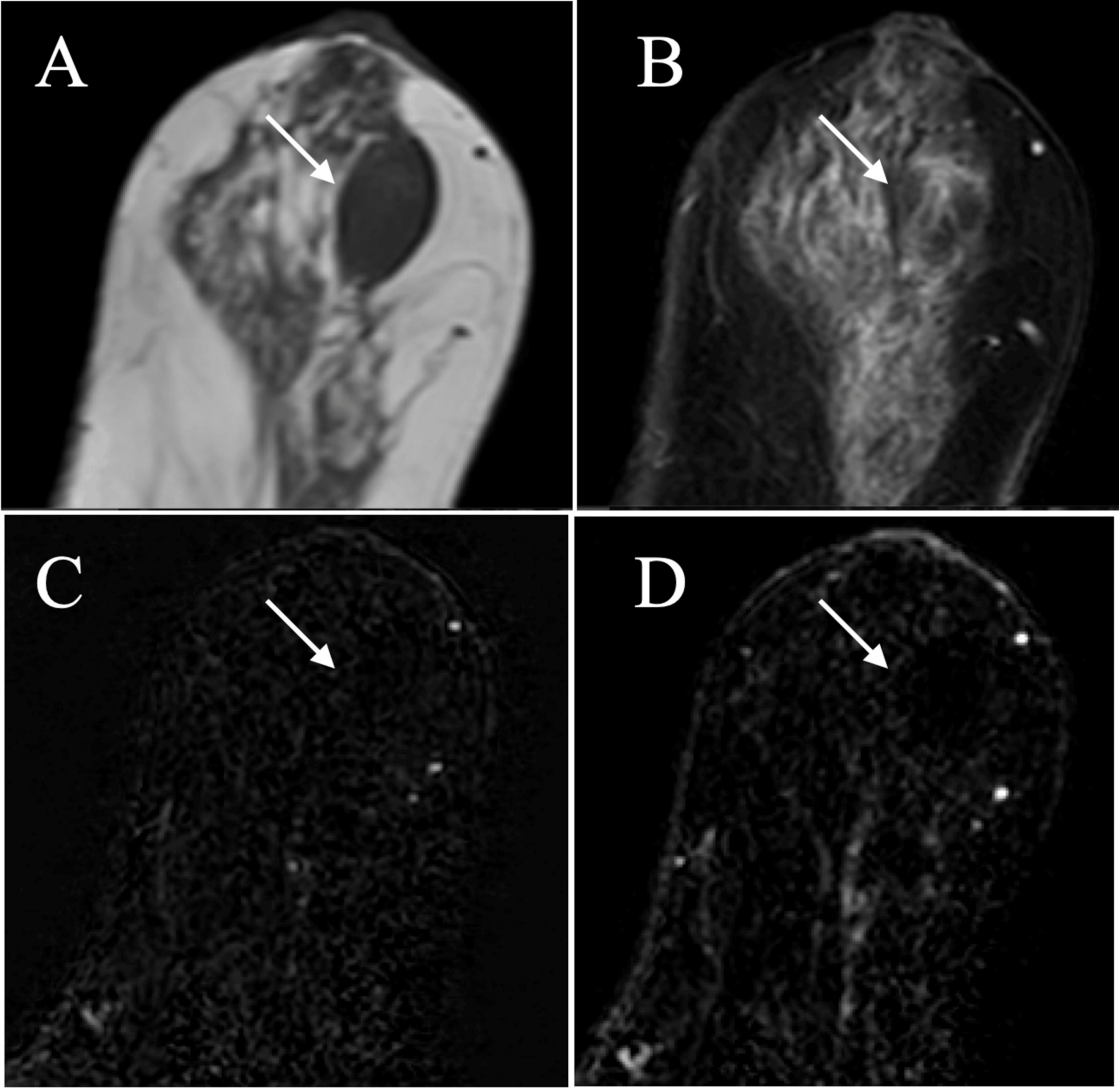
Magnetic resonance imaging (MRI) findings MRI of the breast mass (arrow) showed low signals on T1-weighted images (A), predominant low signals on fat-suppressed T2-weighted images (B), and no enhancement on dynamic studies (C and D).

Magnetic resonance imaging (MRI) of the breast mass showed low signals on T1-weighted images, predominant low signals on fat-suppressed T2-weighted images, and no enhancement through early-to-late-phase images on dynamic studies (Figure 3).

**Figure 4:**
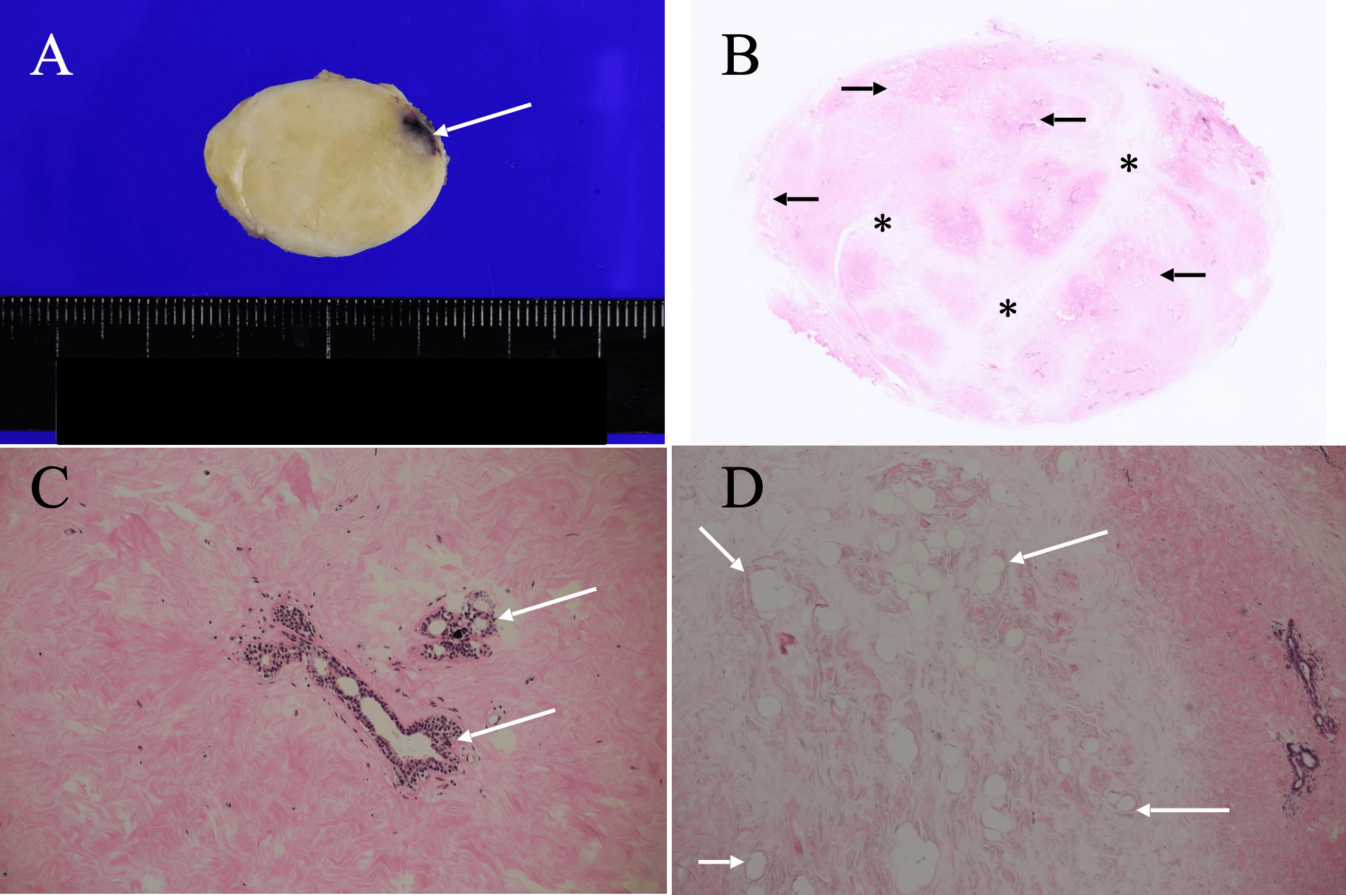
Pathological findings A. The bisected mass was well-circumscribed and whitish except for the black area (arrow), i.e., presumed bleeding, corresponding to the needle biopsy site. B. A low-magnification view showed abundant fibrous components (asterisks) and focal small adipocyte clusters (arrows) in the mass. C. A magnified view showed sparse mammary duct-lobular structures (arrows) in the massive fibrous component areas. D. The magnified view showed sparse adipocyte clusters (arrows) in the limited areas.

These image findings easily made us negate the possible fibroadenoma and phyllodes tumor, but unfortunately led us to no diagnostic suspicion of a specific benign breast disorder. The patient, therefore, underwent a core needle biopsy, which only showed abundant fibrous components without malignant cells and led to no definitive pathological diagnosis. We, therefore, treated the breast mass with a lumpectomy to get both a pathological diagnosis and a cure of the presumed benign breast mass. The bisected mass had clear borders and whitish cut surfaces except for the core needle biopsy-induced hemorrhage areas. Postoperative pathological study showed predominant fibrous components, a small amount of adipocytes, and sparse mammary duct-lobular structures in the fibrous components, leading to the diagnosis of fibrous breast hamartoma (Figure 4).

The patient recovered uneventfully and has been well for two months without any problems in the operated breast.

## Discussion

Breast hamartomas very often have abundant fat and are judged as category 2 on mammography, generally leading to being regarded as out of detailed examination, even though they are large [5]. We, however, evaluated this breast mass as category 3 due to the absence of fat findings on mammography. Despite the absence of malignant findings in the core needle biopsy specimen, no distinct diagnosis was made. We performed a lumpectomy on this breast tumor.

Ultrasound forms mass shapes through the ultrasound wave reflection at the mass borders [6]. In short, much larger interfaces than the ultrasound wavelength at the mass borders, if present, cause ultrasound wave reflection, leading to the depiction of mass margins [7]. Breast hamartomas generally have clear and smooth borders and are generally depicted as oval masses with distinct margins on mammography, as wavelengths actually observed in this case. Large differences in acoustic impedance between the fibrous components in the mass and the fat just adjacent to the mass formed the very clear mass margins with strong high echoes in this case. Mass margins horizontal to the overlying skin showed very strong linear high echoes due to the massive ultrasound wave reflection to the ultrasound probe, whereas oblique margins to the skin presented much weaker signals compared to the horizontal margins due to much less ultrasound wave return to the ultrasound probe. Ultrasound wave returns to the ultrasound probe and also generate the internal high echoes of masses when the ultrasound waves hit the scattering bodies, i.e., much smaller than the ultrasound wavelength, and scatter backward to the ultrasound probe [8]. Adipocytes have very low acoustic impedance, which can cause strong backscattering of the ultrasound waves, make internal echoes of masses high when present within the masses, and were only located as small adipocyte clusters in very limited areas, compatible with the internal high echo areas, in this case.

Mammary glands have three major pathological components: duct-lobular systems, fibrous components, and fat components. Typical breast hamartomas have abundant adipocytes and are easily diagnosed with both mammography and ultrasound [9].

Breast hamartomas, however, need careful differential mammographic and ultrasound diagnosis between fibroadenomas and phyllodes tumors when having abundant fibrous components in this case. As can be easily understood from the imaging findings in this case, MRI findings are extremely useful for the fibrous breast hamartoma diagnosis [10]. Why this breast hamartoma had predominantly fibrous components naturally remains uncertain. To the best of our knowledge, no studies, however, have reported fibrous breast hamartomas to date.

It, therefore, is imperative that diagnostic physicians note the possible fibrous breast hamartomas in the differential diagnosis of presumed benign breast disorders. Breast specialists can accordingly follow up the presumed fibrous breast hamartoma when showing compatible image findings to those observed in this case, without doing unnecessary surgical intervention for this disorder.

To our knowledge, no studies have reported fibrous breast hamartomas to date. Why fibrous breast hamartomas have not been reported naturally remains uncertain. We cannot rule out the possibility that fibrous breast hamartomas actually existed but had simply not been reported. We think that the paucity of reports on fibrous breast hamartomas may be attributable to the inherently low proportion of fibrous tissue within the normal breast.

## Conclusions

Breast hamartomas exclusively have no enhancement on MRI, despite their subtypes. It, therefore, is very important for diagnostic physicians to note the possible fibrous breast hamartomas when having no mass enhancement and presumed predominant fibrous components when performing differential diagnosis of benign breast disorders. In addition, abundant fibrous components and no malignant cells on core needle biopsy can give an important diagnostic clue to diagnostic physicians.
